# Considerations of Low Carbohydrate Availability (LCA) to Relative Energy Deficiency in Sport (RED-S) in Female Endurance Athletes: A Narrative Review

**DOI:** 10.3390/nu15204457

**Published:** 2023-10-20

**Authors:** Melissa T. Lodge, Christie L. Ward-Ritacco, Kathleen J. Melanson

**Affiliations:** 1 Department of Kinesiology, College of Health Sciences, University of Rhode Island, Kingston, RI 02881, USA; christieward@uri.edu; 2 Department of Nutrition, College of Health Sciences, University of Rhode Island, Kingston, RI 02881, USA; kmelanson@uri.edu

**Keywords:** carbohydrates (CHO), relative energy deficiency in sport (RED-S), low energy availability (LEA), low carbohydrate availability (LCA), female athletes, menstrual cycle

## Abstract

The purpose of this narrative review is to identify health and performance consequences associated with LCA in female endurance athletes. The intake of carbohydrates (CHO) before, during, and after exercise has been demonstrated to support sport performance, especially endurance activities which rely extensively on CHO as a fuel source. However, low energy availability (LEA) and low carbohydrate availability (LCA) are common in female athletes. LEA occurs when energy intake is insufficient compared to exercise energy expenditure, and LEA-related conditions (e.g., Female Athlete Triad (Triad) and Relative Energy Deficiency in Sport (RED-S)) are associated with a myriad of health and performance consequences. The RED-S model highlights 10 health consequences and 10 performance consequences related to LEA. The independent effect of LCA on health and performance has been under-researched, despite current CHO intake being commonly insufficient in athletes. It is proposed that LCA may not only contribute to LEA but also have independent health and performance consequences in athletes. Furthermore, this review highlights current recommendations for CHO intake, as well as recent data on LCA prevalence and menstrual cycle considerations. A literature review was conducted on PubMed, Science Direct, and ResearchGate using relevant search terms (i.e., “low carbohydrate/energy availability”, “female distance runners”). Twenty-one articles were identified and twelve met the inclusion criteria. The total number of articles included in this review is 12, with 7 studies illustrating that LCA was associated with direct negative health and/or performance implications for endurance-based athletes. Several studies included assessed male athletes only, and no studies included a female-only study design. Overall, the cumulative data show that female athletes remain underrepresented in sports science research and that current CHO intake recommendations and strategies may fail to consider female-specific adaptations and hormone responses, such as monthly fluctuations in estrogen and progesterone throughout the menstrual cycle. Current CHO guidelines for female athletes and exercising women need to be audited and explored further in the literature to support female athlete health and performance.

## 1. Introduction

Over the past 50 years, female athlete participation has been on the rise, with the Tokyo Olympics in 2020/2021 being the first ever gender-balanced Olympics [1]. However, research focused on the female athlete has not yet corrected for this increased participation in both recreational and elite-level sports. For example, in a review of 1382 exercise and sports science articles published from 2011–2013, 39% of study participants identified as female, and only 4–13% of the articles incorporated a female-only design in their studies [2]. An updated review of six major journals, including a total of 5261 sports science articles published from 2014–2021 illustrated that 34% of all participants reviewed identified as women and 6% of articles included women-only study designs [3]. This highlights the need to balance sports science research on female and male athletes.

Relatedly, the importance of acute carbohydrate (CHO) intake has primarily been studied in male athletes and recommendations have been extrapolated to female athletes. However, it may be necessary to account for sex-based differences in determining CHO intake guidelines based on sex and sport type. In a 2022 publication, researchers conducted an audit of 937 research studies evaluating acute CHO intake recommendations to assess if there was evidence to support the use of these strategies among female athletes [4]. Only 11% (*n* = 197 of 937 studies) of the participants in these studies were women, and of these studies, only 31% utilized sufficient methodologies to define menstrual status [4]. The persisting nutritional recommendations for female athletes emphasize the primary goal of obtaining sufficient energy availability (EA) and hydration. Once caloric needs have been met, the composition of EA can then be considered, including CHO, protein, and fat availability [5]. This lack of high-quality research using female-specific study designs directly impacts our ability to provide high-quality, population-specific nutritional recommendations for athletes.

**Figure 1:**
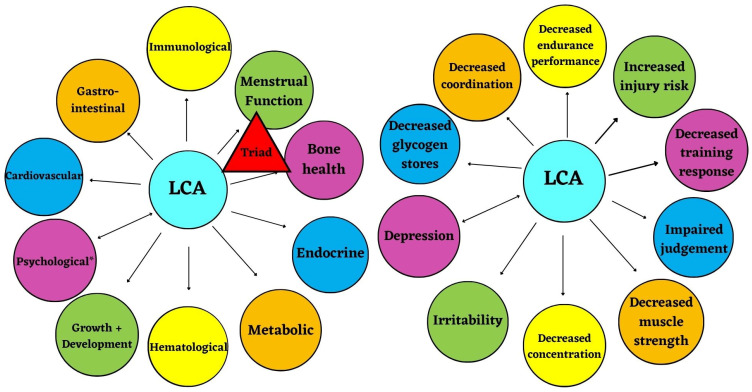
Proposed theoretical model; low carbohydrate availability (LCA) has independent health and performance consequences, illustrated in the Relative Energy Deficiency in Sport (RED-S) model due to low energy availability (LEA) (* Psychological consequences can either precede LCA or be the result of LCA) (adapted from Mountjoy et al., 2014 [6]).

Given that the prevalence of low EA (LEA) is fairly common in female endurance athletes, a growing area of concern is that athletes are not meeting their CHO recommendations. These athletes may experience an additive effect of low carbohydrate availability (LCA) in addition to the health and performance consequences associated with LEA. Figure 1 illustrates the proposed theoretical model of LCA, adapted from Mountjoy et al., 2014, to demonstrate the potential independent effect of LCA on health and performance consequences associated with LEA [6]. The purpose of this narrative review is to evaluate the current evidence that gives rise to the consideration of LCA and the associated health and performance consequences in female endurance athletes.

## 2. Low Energy Availability

The following equation is traditionally used in sports science literature to define energy availability (EA): EA (kcal/kg fat-free mass (FFM)/day) = energy intake (EI; kcal) − exercise energy expenditure (EEE; kcal), normalized to fat-free mass (FFM; kg) [7]. It is often recognized that reduced or subclinical EA ranges from 30–45 kcal/kg FFM/day, which can serve as a tolerable range for athletes seeking to lose weight as part of a diet or exercise program that is short in duration [7]. Typically, LEA is defined as less than 30 kcal/kg FFM/day and illustrates an unsafe energy balance for optimal body function [7]. Physiological responses to the dose and duration of LEA may vary depending on sex, sport type, and genetics [7]. LEA is more common in female athletes compared to male athletes [8]. LEA is associated with unfavorable health and sport performance consequences, as illustrated by LEA-related conditions such as Female Athlete Triad (Triad) and Relative Energy Deficiency in Sport (RED-S) [8,9].

### 2.1. Female Athlete Triad (Triad)

The Female Athlete Triad (Triad) was proposed by the American College of Sports Medicine (ACSM) in a 1992 position statement and updated in 2014 [9,10]. The Triad demonstrates an interrelated condition of (1) LEA with or without eating disorder/disordered eating (ED/DE), (2) osteoporosis, or low bone mineral density (BMD), and (3) functional hypothalamic amenorrhea, or menstrual irregularities. Prospective studies to date illustrate a causal relationship between LEA and menstrual disturbances [9,10,11,12,13,14]. Additionally, current findings show a strong correlation between Triad and negative bone health outcomes, such as increased bone stress injuries and decreased BMD [6,7,9,11,14,15,16,17,18].

### 2.2. Relative Energy Deficiency in Sport (RED-S)

The International Olympic Committee (IOC) introduced the conceptual model of Relative Energy Deficiency in Sport (RED-S) in a 2014 position statement [6]. This conceptual model, which includes consequences of LEA, was updated in 2018 [6,8]. The RED-S conceptual model outlines 10 health consequences (e.g., menstrual function, bone health, hematological, psychological, endocrine, immunological, metabolic, growth and development, cardiovascular, gastrointestinal) and 10 performance consequences (e.g., decreased endurance performance, increased injury risk, decreased training response, impaired judgement, decreased coordination, decreased concentration, irritability, depression, decreased glycogen stores, decreased muscle strength) due to LEA [6,8]. RED-S can affect both female and male athletes but is more prevalent in female athletes and the current literature estimates show that 80% of elite and pre-elite female athletes have at least one symptom of RED-S, compared with 47.7% of male Olympic-level athletes [19,20]. Furthermore, 37% of elite and pre-elite female athletes demonstrated two or more symptoms of RED-S, compared to 15.9% of male athletes [19,20].

### 2.3. The Role of LCA in LEA

When individuals do not consume enough CHO, compared to the requirements for their body composition and physical activity or exercise levels, it can lead to LCA. LCA is a separate concern, independent of LEA, though they often occur simultaneously [7]. LCA is defined as a diet with low CHO intake before, during, or after exercise, which reduces the amount of available CHO for the body to use due to low endogenous (glycogen stores) and/or low exogenous (CHO and glucose intake) levels of CHO [21,22]. Most intervention studies assessing LEA in athletes are also accompanied by a considerable reduction in CHO intake (approximately 25–60% reduction in CHO, depending on the magnitude of LEA) [16,23,24,25]. Therefore, many intervention studies on LEA result in concurrent LCA [20,26,27,28]. This is confirmed by several studies which demonstrate that LCA is common in female athletes who are also at risk for LEA [20,26,27,28].

CHO consumption before, during, and after exercise has been illustrated to benefit sport performance, especially in endurance activities which rely extensively on CHO as a fuel source and substrate for oxidative pathways [29]. CHO substrates provide a greater yield of ATP, or energy, per volume of oxygen delivered to the mitochondria compared to lipids and proteins [29]. Currently, there is no standardized equation or methodology to determine an individual’s CHO availability status as a majority of studies have simply assessed CHO intake. This is a growing area of concern as it has been illustrated that LCA can lead to decreased glucose utilization, hypometabolism, muscular fatigue, impaired fat storage mobilization, decreased performance, and decreased growth hormone production [20,22].

As such, it is important to understand the role of CHO for female endurance athletes and elucidate the health and performance consequences of LCA.

## 3. CHO Background

### 3.1. Physiological and Biochemical Role of Carbohydrates

Under normal conditions, endogenous CHO are stored primarily as glycogen in the skeletal muscle (~500 g) and the liver (~100) [30,31]. The main function of liver glycogen is to maintain blood glucose concentration since the glycogen stored in skeletal muscles cannot be released as glucose due to the lack of glucose 6-phosphatase [30]. Therefore, muscle glycogen is the local energy substrate for exercise, and exercise training can increase the glycogen storage capacity in skeletal muscles, albeit finitely [30,31]. Reduced glycogen content in the skeletal muscle can increase insulin sensitivity, which, in turn, emphasizes the importance of rapidly restoring glycogen content [30].

Muscle glycogen degradation in the myocytes provides the necessary energy for muscle contraction by activating glycogen phosphorylase and debranching enzymes for glycogenolysis [31]. Glycogen phosphorylase activation is dependent on a number of factors to closely regulate the energetic requirements of the working muscles [31]. The allosteric binding of adenosine monophosphate (AMP) and inosine monophosphate (IMP) activates glycogen phosphorylase, which allows the enzyme to be responsive to the energy state of the cell. Furthermore, muscular contractions also increase cytosolic calcium release and an adrenaline-mediated increase in cyclic AMP (cAMP), which activates phosphorylase kinase (PK) and subsequently glycogen phosphorylase [31]. Muscular contractions also activate AMP-activated protein kinase (AMPK) via an increase in cellular AMP/ATP ratios, which results in an inhibition of ATP-utilizing pathways and activation of ATP-generating pathways [31]. Therefore, AMPK promotes glucose uptake, glycolysis, and fatty acid oxidation while inhibiting glycogen and protein synthesis [31]. In mice models, a lack of AMPK notably impaired muscle contraction and reduced voluntary wheel running, which demonstrates the essential role of AMPK in endurance exercise [31]. An increase in calcium concentration in the cells, via phosphorylase kinase activation, stimulates glycogen degradation [31].

The rate of muscle glycogen degradation depends on exercise intensity and training status; as exercise intensity increases, muscle glycogen is depleted at an increasing rate [30,32]. Exercise greater than 70% of VO_2_max utilizes muscle glycogen as the major carbohydrate source. Well-trained individuals improve their ability to metabolize glucose and fat at a much higher capacity compared to untrained individuals [30]. High-intensity activity (e.g., repeated sprinting) can rapidly decrease glycogen stores contained within the active muscle cells, despite the total activity time being relatively brief [32]. However, endurance athletes often train for hours at a time, which causes a marked decline in muscle glycogen over time, especially at exercise intensities greater than 70% VO_2_max [30,32]. Over time, muscle glycogen degradation progressively decreases due to the finite storage of CHO within skeletal muscles [31]. Another critical factor for glycogen metabolism is CHO availability before exercise as increases in the rate of muscle glycogen degradation during exercise are exponentially related to muscle glycogen concentrations before exercise [31].

### 3.2. Metabolism of CHO

Exercise performance typically decreases due to an impaired availability and, hence, ability to use CHO as fuel, which is necessary for exercise at higher intensities and recommended for longer durations such as endurance training [33]. Few studies have attempted to define CHO availability; however, one study calculated CHO availability as the difference between dietary CHO intake and the amount of CHO oxidized during exercise beyond the requirements of normal activity [23]. Nonetheless, LCA ranges have not been established based on these criteria for CHO availability, and many studies continue to report CHO intake, using low CHO intake to define intake below current recommendations [34,35,36,37,38,39,40,41,42,43].

LEA, mediated by reduced CHO availability, affects leptin and triiodothyronine (T3) concentrations, which, in turn, can suppress the resting metabolic rate (RMR) [44]. In two conditions of identical LEA (with one achieved from dietary restriction and the other one achieved from exercise), CHO availability was 57% higher in women who reduced EA via exercise compared to reduced EA via dietary restriction [45]. In this same study, leptin levels decreased by 50% in response to dietary restriction, and this difference is proportional to the reductions in CHO intake [45]. Restricted EA conditions, due to reduced energy intake, of 10 kcal/kg LBM/day, 20 kcal/kg LBM/day, 30 kcal/kg LBM/day, reduced CHO availability by approximately 80%, 60%, and 40%, respectively [23]. In this study, CHO availability was defined as the difference between CHO intake and the amount of CHO oxidized during exercise beyond the requirements of normal activity [23]. These data demonstrate that there is a dose-dependent effect of LEA on CHO availability and that dietary restriction is detrimental to the use of CHO during activity [23].

Additionally, it has been demonstrated that skeletal muscle alters its fuel source in response to LEA conditions, oxidizing less CHO and more fat during exercise [45]. This may have significant effects on athletic performance due to CHO having a faster rate of energy production, compared to fatty acids, to fuel activity. Since glucose metabolized from muscle glycogen cannot enter systemic circulation, CHO availability in the muscles can be adequate while CHO availability systemically is low [5]. Future research should attempt to classify LCA due to low CHO intake based on exogenous (CHO and glucose intake) and endogenous (glycogen stores) availability [22,46].

LCA is common in female athletes, especially those at risk for LEA [20,26,27,28]. Despite no current recommendations for CHO intake before activity specific to female athletes, it is suggested that consuming a high CHO snack (e.g., granola bar, dried fruit, pretzels, and/or fruit juice) 3–4 h before exercise can help mitigate the effects of reduced gluconeogenesis rates during the luteal phase [5].

### 3.3. Current CHO Recommendations

The current recommended amount of CHO for athletes is 55–75% of total calories [5,43]. However, more specific recommendations vary from 5–12 g CHO/kg BW/day for athletes, depending on training activities [33]. Table 1 outlines specific recommendations for CHO intake provided by various organizations and governing bodies. It is important to emphasize the incorporation of small CHO-rich snacks or sports drinks and acute CHO fueling and refueling strategies before and after key training sessions, especially on competition days [29]. Therefore, the current acute recommendation for CHO intake for 4–6 h following a glycogen-depleting exercise session is ≥1.2 g CHO/kg BW/h [5].

### 3.4. Prevalence & Current Intake of CHO

A collection of investigations found that average CHO intake is low in female athletes, compared to current recommendations (discussed above), across various sport types. Fifty percent of female soccer players consumed < 5 g CHO/kg body mass (BM)/day, while 5 g CHO/kg BM/day is the adult sport nutrition recommendation [26]. In a study of female collegiate distance runners, 85% of runners consumed less than the recommended amount of CHO, with an average CHO intake of 4.7 ± 1.9 g CHO/kg BW/day [49]. Another study showed the 3-day average of CHO intake was below 6 g/kg BM/day in 72.7% of young and elite athletes included in a retrospective analysis of nutritional assessments, which is below the 6–10 g CHO/kg BM/day for athletes exercising at moderate to high intensity [20]. In a retrospective study of young and elite female athletes, the prevalence of CHO intake < 4 g/kg BM/day was 49.2% in young female athletes and 33.3% in elite female athletes, and the prevalence of CHO intake < 8 g/kg BM/day was 98.3% in young female athletes and 83.3% in elite female athletes [20]. A 2020 study examining competitive female Australian rules football players demonstrated that 30% of players were at risk for LEA via the Low Energy Availability Female Questionnaire (LEAF-Q), and only 3.7% of athletes met the current CHO requirements for moderate-intensity exercise [50]. A 2018 study compared nutritional intake between preparatory and competitive phases of training in professional female athletes [51]. Forty-five percent and sixty-six percent of participants reported CHO intake below recommended amounts (<5 g/kg BW/day) during the preparatory and competitive phases, respectively [51]. The summary of quantitative findings suggests that 45–98% of female athletes across a variety of sport types are not meeting the current daily CHO recommendation. Few studies were able to qualitatively explain the lack of proper CHO intake, and future research should continue to explore the behaviors and fueling strategies, specifically around CHO intake, in female athletes.

A recent qualitative study explored elite female soccer players’ perceptions of the role of nutrition, specifically CHO, in player development and performance [28]. Results illustrated that there was considerable confusion and misconceptions related to energy and CHO requirements, which stemmed from external pressures due to key stakeholders (e.g., coaches), social media, and the utilization of skinfold as a form of body composition testing [28]. This fear of CHO leads to underconsumption of CHO, despite CHO-fueling strategies being well-documented as beneficial for health and sports performance [28].

## 4. Low Carbohydrate Availability (LCA) Themes

The aim of this narrative review is to examine the health and performance consequences associated with LCA, as well as major considerations for female endurance athletes as they relate to the condition of LCA. The role of CHO and the implications of LCA are discussed in further detail below.

### 4.1. Literature Search

#### 4.1.1. Methodology of Search

A narrative review process was chosen due to the insufficient number of studies examining the impact of low carbohydrate availability on the associated health and performance consequences outlined in the RED-S model. This narrative review allowed for an evaluation of the limited research to date while demonstrating the extensive literature based on LEA prevalence and low CHO intake that is common in this population. Various scientific databases were utilized to search for primary sources, including PubMed, Science Direct, and ResearchGate. The search was conducted using MeSH-compliant keywords to ensure that the gathered literature was relevant to our aim. Keywords such as “low carbohydrate availability” AND “low carbohydrate diet”, “low energy availability” AND “relative energy deficiency in sport”, “female distance runners” AND “female endurance athletes” were employed. The search period was limited to articles published between 15 June 2003 and 15 June 2023 to ensure the currency and appropriateness of the data included in this review. Articles published between 2003 and 2023 were considered if they were primary research articles, published in English, available in full text, and included athletes as participants.

To ensure the appropriateness of the studies included in this analysis, MTL meticulously examined the titles and abstracts of all retrieved manuscripts, and CWR and KM reviewed all included studies. Exclusion criteria were used to filter out studies that utilized outdated data beyond the designated timeframe, studies with unrelated topics that did not align with the specific objectives of this study (e.g., did not use athletes as participants, or did not investigate the role of CHO in health and performance markers), as well as studies not written in English. Once relevant studies were identified, the authors of this review independently extracted information from the selected articles. This rigorous approach helped maintain the quality and reliability of the data included in the studies included within our review. Furthermore, collaborative discussions were conducted among the review authors to synthesize the key findings and prepare the current narrative review. The review authors ensured a comprehensive analysis of the literature and provided a cohesive and informative narrative that addresses the specific objectives of this paper.

#### 4.1.2. Results of Search

Twenty-one articles were identified, and twelve met the inclusion criteria and were included in this review. The identified studies included one cross-sectional study [52], seven longitudinal studies [34,35,36,38,40,43,53], and four randomized control trials [37,39,41,42]. A full summary of the 12 primary research studies included in this review can be found in Supplementary Materials (Supplementary Table S1).

### 4.2. Results

#### 4.2.1. Health Consequences of LCA

CHO availability plays an important role in maintaining normal estrogen and progesterone levels [43]. As estrogen is associated with decreased gluconeogenesis, reduced exogenous CHO intake can compound these unfavorable rates of gluconeogenesis [5]. By maintaining these naturally cycling hormones, CHO helps regulate normal menstrual cycles and bone health maintenance through benefits to hormones (e.g., estrogen, IGF-1, cortisol, growth hormone) influencing BMD [43]. Therefore, LCA is independently associated with poor bone outcomes (e.g., bone stress injuries, decreased bone mineral density (BMD)), even with adequate EA [43].

A clinical, longitudinal investigation of elite male racewalkers with two 6-day phases showed that negative effects on bone health were observed in athletes who consumed either a low CHO, high fat (LCHF) diet or a LEA diet compared to athletes who consumed an adequate CHO + EA diet [43]. However, bone health consequences were more deleterious (e.g., greater decreases in bone formation markers and increases in bone resorption markers) in the LCHF group compared to the LEA group [43]. In male runners, CHO consumption before, during, and after acute training sessions attenuated bone resorption markers, independent of LEA [38]. In a longitudinal study of 10 adult competitive endurance cyclists (n = 4 females), BMD was assessed at 0 months, 5 months, and 10 months. Energy intake and CHO intake were both significantly below the minimum sport nutrition recommendations and low BMD persisted through the 10 months, which illustrates that LCA may be a primary contributor to chronic LEA as low CHO intake drives down total energy intake [34]. CHO intake before, during, and after exercise illustrated positive effects on short-term bone turnover markers in healthy, physically active men ingesting 8% glucose supplements before, during, and after exercise compared to placebo [53]. A study of 30 world-class racewalkers (n = 5 females) assessed 3.5 weeks of LCHF diet followed by a restoration with CHO feeding [40]. Bone remodeling pathways were impaired after short-term LCHF accommodation, and only the bone resorption marker recovered after acute CHO restoration [40]. Additionally, the effects of post-exercise CHO (with protein) are protective for bone health as it decreases bone resorption markers and increases bone formation markers, which creates a positive bone turnover balance [39].

LCA is also associated with depressed immune function [43]. Heikura et al. investigated CHO-specific themes and practices, such as periodic CHO restriction versus training with high CHO availability, with 46 endurance athletes (n = 27 females). Athletes who practiced periodic CHO restriction had a 9% greater risk of illness and injury, compared to athletes who did not practice CHO restriction [52]. Low CHO intake has been associated with micronutrient deficiencies (especially iron and calcium) which have implications on LEA and bone health, respectively [27]. Endocrine changes due to LEA include triiodothyronine (T3) alterations and decreases in leptin, which affect the metabolism and systemic availability of CHO [44]. A short-term clinical trial of 28 male, elite racewalkers assessed several physiological markers in three groups: (1) high CHO, high EA (CON); (2) low CHO, high fat w/ high EA; or (3) LEA [36]. These data showed that short-term, 6-day CHO restrictions led to small, yet unfavorable changes to iron regulation, immune function, and stress responses [36]. CHO-rich diets in international-level racewalkers (n = 31 males, 6 females) exhibited more favorable changes to post-exercise IL-6 and hepcidin, compared to LCHF, which improved iron availability during a 3-week training program [35].

There is a bi-directional association between iron deficiency and LEA. Energy-deficient athletes are at risk for iron deficiency due to decreased dietary intake, impaired absorption, and foot strike hemolysis [5,54]. Additionally, iron deficiency from decreased dietary intake can also promote energy deficiency by shifting ATP production away from oxidative phosphorylation [5,54]. These mechanistic pathways are likely observed under conditions of LCA as well [55]. One study with 12 well-trained male endurance athletes demonstrated that a high-CHO diet for 24 h post-exercise had more favorable outcomes on inflammation and iron regulation than a low-CHO diet [41]. However, another study illustrated no difference between high- and low-CHO diets for markers of pre- and post-exercise inflammation and iron regulation over a 7-day intervention period [42].

**Figure 2:**
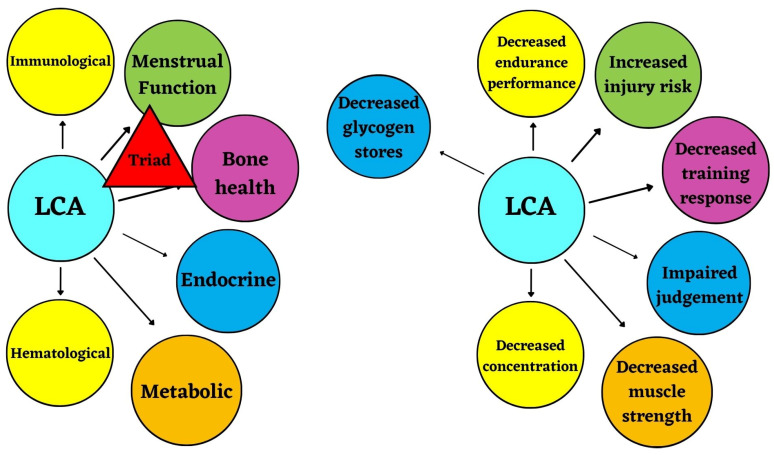
Low carbohydrate availability (LCA) has independent and/or compounding effects on these health (**left**) and performance (**right**) consequences, illustrated in the RED-S model, due to LEA.

Figure 2 and Supplementary Materials illustrate the independent or compounding health consequences due to LCA.

#### 4.2.2. Performance Consequences of LCA

Reduced CHO availability is disadvantageous to exercise performance due to the following pathways: (1) reduced muscle glycogen causes muscular fatigue and a drop in intensity (<60% VO_2_max), and (2) reduced circulating CHO (e.g., blood glucose) for central neural nourishment impairs cognition [5]. In a study of male, endurance-trained cyclists, those consuming a higher protein/lower CHO diet (3.3 g protein/kg BW/d and 5.9 g CHO/kg BW/d) performed significantly worse on a time trial compared to those consuming a diet more representative of the typical endurance athlete diet (1.3 g protein/kg BW/d and 7.9 g CHO/kg BW/d) who saw no significant change in performance from the baseline [37]. Therefore, the endurance cyclists who consumed a higher CHO/lower protein diet, compared to a higher protein/lower CHO diet, performed better, despite similar performance at baseline [37]. Athletes consuming the high CHO diet also illustrated better endurance performance, average power output, and a lower heart rate, compared to those consuming a lower amount of CHO [37]. This may be due to the potential glycogen deficit in the higher protein/lower CHO diet that was compounded by the repetitive effect of maintaining normal training loads [37].

Some studies have demonstrated that low-CHO diets can spare glycogen and enhance fat oxidation, but these adaptations fail to improve performance [33]. Forty-six endurance athletes (n = 27 females) were surveyed regarding training with periodic CHO restriction vs. high CHO availability [52]. Results indicated that 28% and 9% of endurance athletes did not feel that periodic CHO restriction benefited overall training and indicated a greater risk of injury, respectively [52]. LCA increases injury risk, especially bone stress injuries, due to the detrimental effect of LCA on bone health [43]. Increased injury risk decreases athlete availability for training and competition, as well as limiting advantageous performance adaptations to training.

Iron deficiency is a common concern in athletes with LEA and LCA [55,56]. There is currently no consensus in the literature that illustrates strong evidence to suggest that LCA has a direct negative effect on iron regulation or iron status [55]. However, iron intake is lower in athletes consuming a low CHO diet, compared to those consuming CHO-rich diets, thus increasing the risk of iron deficiency [55]. Iron is a necessary nutrient that plays an important role in several athlete-related processes (e.g., O_2_ transport, cellular energy production, immune function, and cognitive processing), which optimizes endurance performance [55]. Negative performance consequences (e.g., decreased endurance performance, decreased training response) are observed in athletes with iron deficiency due to the important role of iron in oxygen delivery and hemoglobin production [55]. Once iron deficiency was corrected via supplementation, increases in VO_2_max, time trial performances, and exercise efficiency were observed in athletes [55].

The independent or compounding performance consequences of LCA outlined here are illustrated in Figure 2 and Supplementary Materials.

## 5. Population Considerations

### 5.1. Male vs. Female Populations

Across the 12 primary research studies included in this narrative review assessing the effects of LCA, there were a total of 197 participants. In total, 21% of participants identified as women while 75% identified as men and 4% did not disclose their sex/gender. Several studies (*n* = 7 studies) included male-athlete-only study designs and no studies included a female-only study design.

To the same effect, a recent review demonstrated that females are underrepresented in research undertaken to develop acute CHO intake recommendations for exercise. Of the 937 studies included (n = 11,202 total participants), 34% of all participants identified as women, and only 6% of studies included women-only designs [3]. There is a need to consider male versus female populations and assess sex-based differences in CHO recommendations, metabolism, and LCA. Of particular concern is how the menstrual cycle may affect changes in CHO metabolism and thus respond to LCA as a moderating factor.

### 5.2. Menstrual Cycle Considerations

LCA is common in female athletes, especially those at risk for LEA [20,26,27,28]. Estrogen, a sex hormone that regulates the female reproductive system, impairs gluconeogenesis [17]. Estrogen peaks during ovulation and has another secondary increase during the luteal phase. Despite no current recommendations for CHO intake before activity specific to female athletes, it is suggested that consuming a high CHO snack (e.g., granola bar, dried fruit, pretzels, and/or fruit juice) 3–4 h before exercise can help mitigate the effects of reduced gluconeogenesis rates during the luteal phase and help protect against associated performance decrements [5].

Furthermore, luteinizing hormone (LH) pulse frequency suggests that LH pulsatility may depend on CHO as opposed to EA, as evidenced by disruption to LH pulsatility at a level of CHO availability between 90–130 g/d of glucose [23,45]. Gluconeogenesis rates are higher in the follicular phase, compared to the luteal phase of the menstrual cycle, at exercise intensities > 50% of VO_2_max [5]. Lower CHO metabolism may result in subsequent performance decrements during the luteal phase compared to the follicular phase. To that end, CHO loading (8.4–9 g CHO/kg BW/day) in the mid-luteal phase has resulted in no change to a small increase (13%) in muscle glycogen, which could potentially enhance performance [57,58]. However, CHO loading during the mid-follicular phase has resulted in a 17–31% improvement in muscle glycogen but not performance [58,59,60].

Women oxidize less CHO and more fat at the same relative intensities of exercise compared to men [5]. This is important to consider as it can significantly limit performance in exercise lasting < 90 min in duration [5]. As exercise intensities increase above ~60–65% VO_2_max, a progressive increase in the relative oxidation of CHO will support the activity [61]. There is some debate regarding strategies to increase reliance on fat oxidation to support physical activity at a range of VO_2_max, but increasing reliance on this pathway has yet to illustrate improvements in performance [61].

### 5.3. Controversy of Low vs. High Carbohydrate Diets

Traditional sports nutrition has approached fueling for endurance training with the promotion of adequate or high CHO intake to promote sufficient CHO availability [62]. However, over the past decade, low carbohydrate or ketogenic diets have become more popular [62]. Testimonials from sportspeople who supposedly adhere to low CHO diets have stoked the flames of the most widely debated sports nutrition topics amongst athletes and key stakeholders in recent years [62,63]. Low CHO diets have demonstrated promising effects on body composition and cardiovascular health [64], but more work is needed to address both health and performance outcomes [63,65]. Results in favor of a low CHO diet for athletes are often challenged due to methodological issues, such as diet adherence, CHO intake thresholds, small sample sizes, short intervention periods, as well as assessment of mechanisms which underpin changes in fuel utilization during exercise [63,66].

Current evidence has yet to detect a clear performance benefit of low CHO diets, especially in highly trained athletes and endurance-based sports [63]. While some studies have illustrated no decrement in low CHO diets in moderate-to-vigorous intensity exercise experiences, these studies have also confirmed decreases in the exercise economy observed in highly trained endurance athletes at greater than 70% of their VO_2_max compared to those with high CHO diets [65]. This is an important distinction as highly trained individuals are seeking to optimize performance across the ranges of VO_2_ workloads [65].

## 6. Limitations

There are several key strengths and limitations to this review that ought to be discussed. While the purpose of this narrative review was to provide a detailed summary of LCA in female endurance athletes and its associated health and performance consequences, a more formal systematic review would add further precision and replicability to the associations described in this paper. In addition, many of the studies used to illustrate deleterious health and performance consequences associated with LCA were performed on male athletes. As such, more intervention-based, longitudinal studies are needed, particularly in female athletes.

## 7. Future Directions

Future research on LCA should aim to establish an appropriate equation to determine CHO availability status, as well as ranges for CHO availability, as exists in the LEA research. In female athletes, EA is defined as greater than or equal to 45 kcal/kg FFM/day, reduced or subclinical LEA ranges from 30–45 kcal/kg FFM/day, and clinical LEA less than 30 kcal/kg FFM/day [7]. To date, much of the current literature classifies CHO intake based on recommendations, rather than CHO availability. LCA has commonly been defined as low CHO intake that reduces the amount of CHO available for the body to use for energy [21,22]. Few studies calculate LCA or use it as a primary outcome, despite it being an important indicator of CHO availability for the body to use, which is of importance to fuel exercise and physical activity. In the current literature, CHO availability was calculated by taking the difference between dietary CHO intake and the amount of CHO oxidation during exercise beyond the minimal requirements of normal physiological function [23]. Perhaps this proposed equation for CHO availability should be adopted across LCA research. Further research is needed to establish a specific equation to determine CHO availability and the appropriate thresholds for adequate, sub-clinically low, and low CHO availability.

It is necessary to audit current guidelines for CHO intake to include sport-specific recommendations and strategies for female athletes to support their health and performance as well as ensure that proper CHO availability is being met through the recommended CHO intake guidelines.

## 8. Conclusions

Women and girls in sports are currently underrepresented in research that has been utilized to develop CHO intake recommendations. Current CHO recommendations and fueling strategies fail to consider female-specific research, adaptations, and fluctuating hormones due to the menstrual cycle or hormonal birth control. Implications of research on LCA will provide more sex-specific guidelines and CHO recommendations that prioritize a proper ratio of macronutrients for optimal functioning. Coaches, athletes, sports dietitians, and other members of the athlete’s care team would be able to use these recommendations and strategies to help support the health and performance of the female athlete. In conclusion, diets with adequate energy and CHO availability support the physiological demands of elite athletes while minimizing unfavorable health and performance consequences.

## Figures and Tables

**Table 1:** Carbohydrate intake recommendations by source (e.g., organization or governing body) for female athletes [5,47,48].

Source	Energy Intake or Energy Availability in Female Athletes	Carbohydrate Intake in Female Athletes
American College of Sports Medicine (ACSM), Academy of Nutrition and Dietetics (AND), and Dietitians of Canada (DC)	Adequate EA ≥ 45 kcal/kg FFM/day LEA < 30 kcal/kg FFM/day	6–10 g CHO/kg BW/day (athletes)
Recommendations and Nutritional Considerations for Female Athletes: Health and Performance	Adequate EA ≥ 45 kcal/kg FFM/day Subclinical LEA 30–45 kcal/kg FFM/day LEA < 30 kcal/kg FFM/day	7–10 g CHO/kg BW/day
International Olympic Committee (IOC)	Adequate EA ≥ 45 kcal/kg FFM/day Subclinical LEA 30–45 kcal/kg FFM/day LEA < 30 kcal/kg FFM/day	5–7 g CHO/kg BW/day (moderate exercise program, ~1 h/day); or 6–10 g CHO/kg BW/day (endurance program, moderate to high intensity, 1–3 h/day)
International Society of Sports Nutrition (ISSN)	N/A	5–8 g CHO/kg BW/day (moderate- to high-intensity volume, 2–3 h/day, 5–6 times a week); or 8–10 g CHO/kg BW/day (high volume, intense exercise, 3–6 h/day, 1–2 sessions, 5–6 times a week)
2020–2025 Dietary Guidelines for Americans Advisory Committee	EI: 2000–2400 kcal/day (females, moderately active to active, aged 18–50 years)	45–65% of total calories from carbohydrates (females, aged 18–50 years)

Abbreviations: energy intake (EI), energy availability (EA), carbohydrate (CHO), kilocalories (kcal), body weight (BW), kilogram (kg), fat-free mass (FFM), low energy availability (LEA), hours (h).

## Data Availability

No new data were created or analyzed in this study. Data sharing is not applicable to this article.

## Supplementary Materials

The following supporting information can be downloaded at: https://www.mdpi.com/article/10.3390/nu15204457/s1, Table S1: Summary of 12 primary research studies included in review of the current literature.
