# Supplementary material for: Considerations of Low Carbohydrate Availability (LCA) to Relative Energy Deficiency in Sport (RED-S) in Female Endurance Athletes: A Narrative Review
Source: Nutrients. 2023 Oct 20;15(20):4457. doi: 10.3390/nu15204457 (PMC10609849; doi:10.3390/nu15204457)
Supplement: Supplementary file 1 [file nutrients-15-04457-s001.zip › nutrients-2637227-supplementary.pdf]

Supplementary Materials:

Table S1. Summary of 12 primary research studies included in review of current literature.

ARTICLE	YEAR	AUTHOR (S)	STUDY DESIGN + POPULATION (n)	METHODS (MEASURES/MARKERS) OF INTEREST	INTERVENTION/DURATION	ASSESSMENT OF CARB	RESULTS	BRIEF CONCLUSION	KEY POINT #1	KEY POINT #2	TAKEAWAY MESSAGE
A Mismatch Between Athlete Practice and Current Sports Nutrition Guidelines Among Elite Female and Male Middle- and Long-Distance Athletes	2017	Heikura et al.	cross-sectional, online questionnaire; female and male Canadian, Australian, and American world class middle- (n = 16 females, n = 13 males) and long-distance (n = 11 females, n = 4 males) runners and race walkers (n = 4 males); 18-4yrs., 1050+ IAAF points	questionnaire of on nutritional practices of elite endurance athletes: dietary intakes and nutrient periodization around training sessions, comparison of EA, hormone conc., BMD, and Hb mass changes during altitude training	between- and within-day dietary periodization; 6 themes included whether athletes purposefully eat more or fewer foods, or specific macronutrients on hard or easy training days, within-day questions considered timing of meals around pre- and post-key training sessions, training in fasted state, practices of periodic CHO restriction, and training with high CHO availability	2 CHO-specific themes: practices of periodic CHO restriction, and training with high CHO availability	participants: 27 females (25.9 ± 3.2 yr., 1.68 ± 0.07 m, 53.9 ± 7.3 kg, IAAF score 1113 ± 37 points) and 19 males (27.7 ± 4.5 yr., 1.81 ± 0.06 m, 66.8 ± 658 kg, IAAF score 1114 ± 47 points); 37% of females and males, middle and long-distance (41 vs 32%) athletes reported eating more food on hard training days or the following day, 0% and 26% of long- vs middle-distance athletes reported eating more at main meals, and 37% of females and 5% of males reported eating more within 4h before training; only 9% reported intentional use of CHO restriction, while 11% said they periodically restrict CHO intake	there are current mismatches between athlete practice and current and developing sports nutrition guidelines; particularly with respect to CHO availability	CHO restriction has potential side effects: marathon runners reported feeling that CHO restriction does not benefit training overall (28%), and leads to greater risk of illness and injury (9%)	76% of the athletes mostly because of concerns around poor training quality or the experience of a lack of benefit to performance	endurance athletes may already have occasional, unintentional depleted glycogen stores, due to inability of normal glycogen synthesis rates to restore the glycogen utilization patterns because of high volume/high intensity training; training with further deliberately low CHO availability may offer little extra benefit when balanced against the potential negative health/performance consequences
Energy availability and dietary patterns of adult male and female competitive cyclists with lower than expected bone mineral density.	2015	Viner et al.	short-term longitudinal; 10 adult (29–49 years) male (n = 6) and female (n = 4) competitive endurance cyclists (5 road, 5 off-road), with lower-than-expected BMD	BMD at 0 months/5 months/10 months, EI and EEE during preseason (PS), competition (C), and off-season (OS); Three-Factor Eating Questionnaire (TFEQ)	LEA and dietary patterns	CHO, protein, fat, and energy estimated from 3-day dietary records	70% had LEA (< 30 kcal/kg FFM/day) during PS, 90% during C, and 80% during OS (range: 3–37 kcal/kg FFM/day); 90% of cyclists had LEA during ≥ 1 training period, and 70% had LEA across the season; mean carbohydrate intake was below sport nutrition recommendations during each training period (PS: 3.9 ± 1.1 g/kg/day, p < .001; C: 4.3 ± 1.4 g/kg/day, p = .005; OS: 3.7 ± 1.4 g/kg/day, p = .01)	high prevalence of low EI, and subsequently LEA, in male and female competitive road and off-road cyclists; it's suspected that low carbohydrate intake drives the low EI and LEA	CHO intake was significantly below minimum sport nutrition recommendations	low BMD was maintained through 10mos.	LCA is main contributor in cases of chronic LEA
Six Days of Low Carbohydrate, Not Energy Availability, Alters the Iron and Immune Response to Exercise in Elite Athletes.	2022	McKay et al.	clinical trial, short-term longitudinal; male elite racewalkers (28)	VO2 max, 10km PB, baseline/adaptation training vol., body mass, CRP, testosterone, cortisol, T/C ratio, total cholesterol, HDL, LDL, triglycerides, TC-HDL ratio, ferritin, iron, transferrin, transferrin saturation, hepcidin, hemoglobin, hematocrit, reticulocytes, RBCs	training camp: 2, 6-day phases; phase 1: high carb/high EA diet (CON; ~65% of total energy intake, high EA (>40 kcal/kg FFM), phase 2: i. CON, ii. low carb/high fat w/ high EA (LCHF; same EA, <50 g/day carb, 2.2 g/kg/day protein, ~80% fat.), or iii. LEA (~15 kcal.kg FFM, 60% carbohydrate, 25% protein, 15% fat)	EA = EI - EEE; normalized to FFM (via DEXA); via 1) provision and supervision of a rigorously controlled dietary plan, 2) individualization of EEE calculations based on the athlete's prospective training plan, and 3) real-time manipulation of EI over the day according to actual training completed.	significant interaction effects for triglycerides (p=0.004), as there was a significant decrease in LCHF following adaptation (p=0.041); decreased testosterone following adaptation in all groups (p < 0.001); LCHF had higher hemoglobin conc. following adaptation compared to CON; decrease in reticulocytes in LCHF (-20%; p=0.051), e no changes in CON (+7%; p=0.658) or LEA (+5%; p=0.889); LCHF was significantly slower in 25km TT during adaptation compared to baseline (+7 min 28 sec; p<0.001); post-exercise IL-6 conc. significantly higher after adaptation compared to baseline (26.6 vs. 12.4 pg·mL-1; p=0.019), post-exercise increase in IL-6 levels were greater in LCHF following adaptation compared to CON (+13.1 pg·mL-1; p=0.010); hepcidin conc. increased 3h post-exercise in all groups (p<0.001; largest within LCHF (p=0.011)), and were significantly associated with resting ferritin levels (p<0.001); post-ex increase in lymphocyte and monocytes was largest in LCHF at adaptation compared to baseline (p<0.001); cortisol levels increased in LCHF post-ex (+321 nmol/L; p<0.001) and 1h post-ex (+283 nmol/L; p<0.001) during adaptation compared to baseline; reduction in glucose post-exercise during adaptation (p=0.05).	short-term ketogenic, LCHF diet results in acute perturbations to both the immune and iron regulatory response to exercise.	short-term CHO restriction = small, yet unfavorable, changes to iron, immune, and stress response to exercise in male endurance athletes	no significant negative adaptations to male athlete health were observed with restricted EA compared to adequate EA	LCHF negatively affected several physiological markers of health and performance, compared to CON or LEA
Short-Term Carbohydrate Restriction Impairs Bone Formation at Rest and During Prolonged Exercise to a Greater Degree than Low Energy Availability.	2022	Fensham et al.	clinical trial, short-term longitudinal; male international/national racewalkers (28)	DEXA, RMR, economy, fasting CTX, P1NP, gla-OC, glu-OC	training camp: 2, 6-day phases; phase 1: high carb/high EA diet (CON; ~65% of total energy intake, high EA (>40 kcal/kg FFM), phase 2: i. CON, ii. low carb/high fat w/ high EA (LCHF; same EA, <50 g/day carb, 2.2 g.kg/day protein, ~80% fat.), or iii. LEA (~15 kcal.kg FFM, 60% carbohydrate, 25% protein, 15% fat)	EA = EI - EEE; normalized to FFM (via DEXA); three groups: CON (high CHO, high energy), LCHF (low CHO, high fat), or LEA	all groups increased in fasting CTX; LCHF decreased in fasting P1NP (~26%), glaOC (~22%), + glu-OC (~24%), compared to CON; LEA had smaller reductions in P1NP (~14%) + glu-OC (~24%); no significant differences b/n LCHF + LEA were illustrated	both low carbohydrate + EA may impair energy metabolism, via reduced endocrine action of OC	BTMs provide insight into short-term bone quality changes + may predict fracture risk	negative effects of bone were observed in LCHF and LEA groups, but consequences were more deleterious in LCHF group	diet with adequate energy + carbohydrate to support training in elite athletes seems to prevent unfavorable imbalance b/n bone resorption + formation markers, & may improve energy metabolism

A Whey-Supplemented, High-Protein Diet Versus a High-Carbohydrate Diet: Effects on Endurance Cycling Performance	2006	Macdermid et al.	randomized control trial; competitive endurance cyclists (n = 7)	weight, BP, RMR, lactate and blood glucose, endurance cycling performance, self-paced for a body weight dependent (60 kJ/bm) amount of work (total workload, time, and mean power output), VO2, RER, N excretion	two, separate 7-d	ingested either H-CHO (7.9 ± 1.9 g/kg/d CHO; 1.2 ± 0.3 g/kg/d fat; 1.3 ± 0.4 g/kg/d protein) or H-Pro (4.9 ± 1.8 g/kg/d; 1.3 ± 0.3 g/kg/d; 3.3 ± 0.4 g/kg/d) diet in a randomized, balanced order	performance was significantly (P = 0.010) impaired following H-Pro (153 ± 36) compared with H-CHO (127 ± 34 min), average power output was significantly lower during the H-Pro trials (293 ± 64 and 197 ± 55 W), P = 0.013; RER (0.90 ± 0.11 and 0.85 ± 0.09) and HR (58 ± 13 and 53 ± 10 bpm) were significantly lower following 7d of H-Pro diet (P = 0.036 and P = 0.044); no effect of diet condition on body weight, BP, blood glucose or lactate, hematocrit, RMR;	7-d H-Pro diet had significant overall ergolytic effect on performance when compared to the diet recommended for an endurance cyclist	high CHO diet translated to better endurance performance compared to high-Pro	no differences between treatments were observed for physiological measures, which could possibly explain endurance performance	diet with adequate-high CHO intake supports endurance performance
Post-exercise carbohydrate and energy availability induce independent effects on skeletal muscle cell signalling and bone turnover: implications for training adaptation	2019	Hammond et al.	longitudinal study; male runners (n = 9)	muscle glycogen (via muscle biopsy), p38MAPK phosphorylation, PGC-1α, p53, CPT1 mRNA, P1NP, βCTX, insulin, leptin, ghrelin, adiponectin, β-OHB, NEFA, glycerol, lactate,	morning (AM) and afternoon (PM) high-intensity interval (HIT) (8 × 5 min @ 85% VO2peak) running protocol (interspersed by 3.5h) under dietary conditions	3 dietary conditions: (1) high CHO availability (HCHO: CHO ~12 g/kg, EA ~60 kcal/kg FFM), (2) reduced CHO but high fat availability (LCHF: CHO ~3g/kg, EA 60 kcal/kg FFM) or (3), reduced CHO and reduced EA (LCAL: CHO 3 g/kg, EA 20 kcal/kg FFM)	CHO oxidation was significantly greater during PM HIT in HCHO compared to LCHF (P = 0.001) and LCAL (P = 0.004); lipid oxidation was significantly greater during LCHF (P = 0.014) and LCAL (P = 0.011) compared to HCHO; plasma glucose was significantly higher in HCHO compared to LCHF (P < 0.001) and LCAL (P = 0.003), restricting CHO and energy intake post-exercise induced significantly greater plasma NEFA and β-OHB in LCHF (P = 0.001 and 0.001) and LCAL (P = 0.007 and 0.001); β-OHB was also different (P = 0.024) between LCHF and LCAL; plasma insulin conc. was significantly higher (all P < 0.01) immediately, 3 h and 17 h post exercise in HCHO compared to LCHF and LCAL; no apparent differences in muscle glycogen were illustrated between HCHO, LCHF and LCAL at after PM HIT; muscle glycogen re-synthesis was observed such that glycogen conc. was higher in HCHO compared to both LCHF (P = 0.028) and LCAL (P = 0.002) at 3h and 17h post exercise; mRNA expression of SIRT1 significantly greater in LCAL compared to HCHO (P = 0.013) at 17h post-ex; βCTX concentration was significantly lower immediately before, immediately after, and 3h after PM HIT in HCHO compared to LCHF (P = 0.032) and LCAL (P = 0.035)	in recovery conditions where muscle glycogen conc. remains within range of 200–350 mmol/kg dw, acute post exercise CHO and energy restriction (e.g., < 24 h) does not potentiate activation of key cell signaling responses that are associated with the regulation of hallmark adaptations to endurance training	reduced CHO availability, LEA, or both before and after exercise may augment endurance training-induced adaptations of human skeletal muscle, as mediated via modulation of cell signaling pathways	CHO consumption before, during and after an acute training session attenuated markers of bone resorption, effects that are independent of EA	low CHO availability illustrated independent effects of LEA via negative bone adaptations (e.g., increasing bone resorption)
Chronic Adherence to a Ketogenic Diet Modifies Iron Metabolism in Elite Athletes	2019	McKay et al.	longitudinal study; international-level race walkers (n = 50 athlete data sets; n = 37 athletes, 31 males, 6 females)	19- to 25-km race walking test protocol at baseline and after adaptation; serum ferritin, IL-6, and hepcidin conc.	3-wk. periodized training program	three dietary interventions: (i) a high-CHO diet, (ii) a periodized CHO availability, or (iii) an LCHF diet; high-CHO and periodized CHO were combined into one group for analysis	decrease in serum ferritin across intervention period was substantially greater in CHO group (37%) compared to LCHF (23%) group (P = 0.021); after dietary intervention, postexercise increase in IL-6 was greater in LCHF (13.6-fold increase) than CHO-rich diet (7.6-fold increase; P = 0.033); no significant differences occurred between diets, CI values indicate that 3h postexercise hepcidin conc. were lower after intervention compared with baseline in CHO, with no differences evident in LCHF.	LCHF diet athletes should routinely monitor their iron status in combination with markers of inflammation, to determine changes to iron availability	CHO-rich diet exhibited more favorable changes to postexercise IL-6 response, compared to LCHF group	CHO-rich diet exhibited more favorable changes to postexercise hepcidin response, compared to LCHF group	more favorable inflammatory response to exercise (IL-6; as influenced by an athlete's glycogen status) and iron-regulation (via increased hepcidin, promotes movement of iron via iron export transporter, ferroportin, enhancing amount of iron absorbed from diet and recycled by macrophages) in CHO-rich diets
Effect of carbohydrate feeding on the bone metabolic response to running	2015	Sale et al.	longitudinal study; healthy, physically active men (n = 10)	120-min treadmill run at 70% of VO2 max; markers of bone resorption (βCTX) and bone formation (P1NP), osteocalcin (OC), parathyroid hormone (PTH), albumin-adjusted calcium (ACa), phosphate, GLP-2, IL-6, insulin, cortisol, leptin, osteoprotogerin (OPG)	Two, 7-day trials	two groups: fed CHO (8% glucose immediately before, every 20 min during, and immediately after exercise @ rate of 0.7 g CHO/kg BM/hr.) and once being fed placebo (PBO)	CHO feeding exhibited significantly lower βCTX, P1NP, and IL-6 responses in immediate postexercise period compared with PBO (βCTX: P = 0.028; P1NP: P = 0.021; IL-6: P = 0.036); no difference in the short-term response; significant effects of exercise (PBO trial) were shown for insulin (P < 0.001), IL-6 (P < 0.001), and leptin (P < 0.01), no significant effects of exercise on cortisol, OPG, or GLP-2; CHO feeding significantly attenuated increase in IL-6 conc. (223%) seen immediately after exercise in the PBO trial, not observed over follow-up days	CHO supplementation during prolonged running reduced bone turnover in the hours following exercise; IL-6 was a possible mediator of immediate bone resorption response to exercise with CHO feeding	CHO feeding during exercise attenuated βCTX and P1NP responses in the hours but not days following exercise, illustrating the acute effect of CHO feeding on bone turnover	IL-6 responded in a similar manner to bone turnover after CHO feeding during exercise	CHO feeding before, during, and after exercise has a positive on short-term bone turnover markers
A Short-Term Ketogenic Diet Impairs Markers of Bone Health in Response to Exercise	2020	Heikura et al.	longitudinal study; world-class racewalkers (n = 30; 25 males, 5 females)	bone breakdown (CTX), bone formation (P1NP), bone metabolism (OC); hybrid laboratory/field test of 25 km (males) or 19 km (females) @ 75% VO2 max	3.5wk.; with a follow-up of high-CHO availability	3.5-week ketogenic low-carbohydrate, high-fat (LCHF) diet and subsequent restoration of carbohydrate (CHO) feeding	after adaptation, LCHF increased fasting CTX concentrations above baseline (p = 0.007), while P1NP (p < 0.001) and OC (p < 0.001) levels decreased; post-exercise, LCHF increased CTX concentrations above baseline (p = 0.001) and above HCHO (p < 0.001), while P1NP (p < 0.001) and OC concentrations decreased (p < 0.001) during exercise; exercise-related AUC for CTX was increased by LCHF after adaptation (p = 0.001), with decreases in P1NP (p < 0.001) and OC (p <	long-term health effects of this LCHF intervention are largely unknown	bone modeling/remodeling markers were impaired after short-term LCHF diet	only the bone resorption marker recovered after acute CHO restoration	adequate CHO availability is protective against negative consequences of bone turnover observed in LCA diets

							0.001); CHO restoration recovered post-exercise CTX and CTX exercise-related AUC				
The Effect of Postexercise Carbohydrate and Protein Ingestion on Bone Metabolism	2017	Townsend et al.	randomized, counterbalanced, placebo-controlled, and single-blinded crossover study; trained young men (n = 10)	βCTX, P1NP, parathyroid hormone, PO4, ACa, Ca, VO2 max	three trials: (i) placebo (PLA), where PLA solution was ingested immediately and 2 h postexercise; (ii) immediate feeding (IF), where CHO + PRO (1.5 g/kg BM dextrose and 0.5 g/kg BM whey) was ingested immediately postexercise and PLA 2 h postexercise; and (iii) delayed feeding (DF), where PLA was ingested immediately postexercise and CHO + PRO solution 2 h postexercise	3-day food diary; diet consisting of 55% CHO, 30% fat, 15% PRO, and isocaloric with habitual diets using dietary analysis software	at 1 and 2 h postexercise, A-CTX conc. were lower in IF trial compared with DF and PLA trials (P < 0.001); @ 3 h postexercise, A-CTX conc. were higher in PLA trial compared with IF (P < 0.001) and DF trials (P = 0.026); @ 4 h postexercise, A-CTX concentrations were lower in DF trial compared with IF (P = 0.003) and PLA trials (P < 0.001); @ 4 h postexercise, P1NP was higher in IF trial compared with DF (P = 0.026) and PLA trials (P = 0.001); @ 3 h postexercise, parathyroid hormone was higher in IF trial compared with DF trial (P < 0.001)	main findings: 1) ingestion of CHO + PRO solution (1.5 g/kg BM of CHO and 0.5 g/kg BM of PRO) suppressed A-CTX concentrations after an exhaustive run, with greater overall suppression when CHO + PRO solution was ingested immediately; 2) immediate ingestion of CHO + PRO resulted in small increases in P1NP conc. @ 3 and 4h postexercise; & 3) delayed ingestion of CHO + PRO solution (2h postexercise) also resulted in a large suppression of A-CTX concentrations	immediate ingestion of CHO + PRO may be beneficial; decreases bone resorption marker and increases bone formation marker, creates a more positive bone turnover balance	rapid response to ingestion is important because elite athletes habitually train multiple times a day, emphasizing the importance of timing fuel and recovery	post-exercise CHO (w/ protein) ingestion is protective for bone health
Acute dietary carbohydrate manipulation and the subsequent inflammatory and hepcidin responses to exercise	2015	Badenhorst et al.	randomized crossover study; well-trained recreational endurance male athletes (n = 12)	VO2max via graded exercise test (GXT), IL-6, hepcidin-25, iron, ferritin, blood lactate, RPE, HR, distance, time training load	3 separate running sessions at the lab (i. GXT, ii. glycogen depletion run, and iii. interval running task)	24-hr. controlled CHO diet: high CHO (10 g CHO/kg BM/day) vs. low CHO (3 g CHO/kg BM/day)	IL-6 was elevated immediately post-exercise compared to baseline in both conditions (p < 0.001), but was lower in HCHO (p = 0.015); hepcidin was also lower at baseline (p = 0.049) in HCHO, and a large effect (d = 0.72) indicated a trend for lower levels at 3-h post-exercise, compared to LCHO; iron increased post-exercise for both trials (p = 0.001), but ferritin remained unchanged pre- to post- or between groups	after completion of a glycogen-depleting (by 50%) exercise task that has previously been established to reduce, high-CHO consumed for 24-h attenuated post-exercise inflammatory response and hepcidin response	effect of low CHO diet on IL-6 and hepcidin levels may negatively impact an athlete's iron metabolism	CHO consumption did not affect ferritin levels in endurance-trained athletes	high-CHO diet for 24-hr post-exercise had more favorable outcomes on inflammation and iron regulation than low CHO
Seven days of high carbohydrate ingestion does not attenuate post-exercise IL-6 and hepcidin levels	2015	Badenhorst et al.	randomized controlled trial; well-trained recreational endurance male athletes (n = 12)	VO2max via graded exercise test (GXT), IL-6, hepcidin-25, iron, ferritin, RPE, HR, distance, time training load	Two, 7-day training blocks consisting of 5 running sessions on days 1, 2, 4, 5, and 7; intensity and duration matched between training blocks	High CHO (8 g CHO/kg BM/day) vs. low CHO (3 g CHO /kg BM/day); each group had isoenergetic diets	immediate postexercise IL-6 were significantly elevated (p = 0.001) from pre-exercise to days 1 and 7; 3-h post-exercise serum hepcidin levels from pre-exercise to days 1 and 7, neither marker was significantly different between groups (low- vs. high-CHO group)	high vs. low CHO diet over a seven-day training program does not significantly affect pre- and post-exercise inflammatory and hepcidin responses markers in response to endurance training	Immediate inflammatory response to exercise did not differ between low- vs. high CHO consumption in endurance-trained athletes	CHO consumption did not affect iron regulation in endurance-trained athletes	CHO consumption did not significantly differ between high- and low- diets for markers of pre- and post-exercise inflammation and iron regulation

Key: carbohydrate (CHO), protein (PRO), energy availability (EA), low energy availability (LEA), low carbohydrate availability (LCA), energy intake (EI), exercise energy expenditure (EEE), low carbohydrate, high fat (LCHF), bone mineral density (BMD), body mass (BM), area under curve (AUC), C-terminal telopeptide (A-CTX), C-terminal cross-linked telopeptide of type I collagen (βCTX), amino-terminal propeptide of type 1 procollagen (P1NP), interleukin-6 (IL-6), albumin-adjusted Ca (ACa), high-density lipoprotein (HDL), low-density lipoprotein (LDL), maximum volume of oxygen consumption (VO2 max), rating of perceived exertion (RPE), dual-energy x-ray absorptiometry (DEXA), resting metabolic rate (RMR).

*Green cells = positive physiological effect of LCA; Yellow cells = neutral/no physiological effect of LCA; Red cells = negative physiological effect of LCA
